# Domestication and Feed Restriction Programming Organ Index, Dopamine, and Hippocampal Transcriptome Profile in Chickens

**DOI:** 10.3389/fvets.2021.701850

**Published:** 2021-09-16

**Authors:** Siyu Chen, Chao Yan, Jinlong Xiao, Wen Liu, Zhiwei Li, Hao Liu, Jian Liu, Xiben Zhang, Maojun Ou, Zelin Chen, Weibo Li, Xingbo Zhao

**Affiliations:** ^1^Guangdong Provincial Key Laboratory of Animal Molecular Design and Precise Breeding, Key Laboratory of Animal Molecular Design and Precise Breeding of Guangdong Higher Education Institutes, School of Life Science and Engineering, Foshan University, Foshan, China; ^2^Guizhou Nayong Professor Workstation, China Agricultural University, Bijie, China; ^3^College of Animal Science and Technology, China Agricultural University, Beijing, China

**Keywords:** domestication, feed restriction, dopamine, hippocampus, chicken

## Abstract

The domestication process exerts different phenotypic plasticity between slow- and fast-growing breeds of chicken. Feed restriction has a critical role in production performance, physiological plasticity, and stress response. Our study aimed to explore how feed restriction programed the organ index, dopamine, and hippocampal transcriptome profile between slow- and fast-growing chickens, which were fed either *ad libitum* (SA and FA), or feed restricted to 70% of *ad libitum* (SR and FR), for 30 days. Results showed that feed restriction influenced the brain organ index (*P* < 0.05), but not the organ index of the heart, liver, and spleen. The slow-growing breed tested had a higher brain organ index than the fast-growing breed (*P* < 0.05). Under feed restriction conditions, both the slow- and fast-growing breeds had significantly elevated dopamine concentrations (*P* < 0.05) compared to those fed *ad libitum*. In the GO term, upregulated genes in the FA group were enriched in the mitochondria, respiratory chain, and energy metabolism compared to the SA group (*P* < 0.05). Membranes and ribosomes were enriched in the cellular component between the SR and FR groups (*P* < 0.05). In the KEGG functional pathways, upregulated DEGs in the FR group were enriched in the cardiovascular disease category and neurodegenerative disease category compared to the FA group (*P* < 0.05). Downregulated DEGs in the FA group were enriched in the oxidative phosphorylation and neurodegenerative disease categories (Parkinson's disease and Huntington's disease) compared with the SA group (*P* < 0.05). Upregulated DEGs in the FR group were enriched in the cardiovascular disease category, neurodegenerative disease category, and energy metabolism than the SR group (*P* < 0.05). In conclusion, feed restriction had profound effects on the brain organ index and plasma dopamine in the slow- and fast-growing chickens. Feed restriction may result in issues relating to cardiovascular and neurodegenerative diseases in the fast-growing breed tested, but not in the slow-growing breed.

## Introduction

Modern chickens, the descendants of the Red Junglefowl, have undergone basic changes, including to behavior and reproduction (1–3), as well as in brain morphology, gene expression, and DNA methylation as compared to their ancestors (4). Of which, the fast-growing breeds under intensive domestication were directly selected for meat in order to meet market demands in the past decades (5). Fast-growing chickens have achieved the intended beneficial effects to meet the needs of humans, which results in animal welfare problems and compounding of undesirable traits in response to intensive selection. For example, over-feeding and rapid growth cause cardiovascular disease, skeletal burden, and metabolic stress (5, 6), as well as immune function and parent stock management challenges in broiler chickens (7). On the other hand, most Chinese native chicken breeds are dual-purpose breeds that grow slowly under less intensive domestication processes, with divergent phenotypes from fast-growing modern breeds (8). Existing evidence has proven that slow- and fast-growing breeds have undergone phenotypic changes relating to leg muscle gene expression (9), production performance (10), as well as stress resistance (11). Notably, feed restriction programming—the restriction of nutrient intake by limiting the growth rate—was widely applied in the poultry industry. Animals under a long-term period of starvation suffer chronic stress.

Accordingly, the timing, duration, and intensity of feed restriction comprehensively influence the growth, physiological phenotypes, behavior (12, 13), and stress response of birds (14). For example, feed restriction to 90% from 5- to 11-days-old resulted in higher body weight and superior capability for meat production than feed restriction to 70% from 5- to 18-days-old, compared to feed free intake group. Likewise, early-stage (from 5 days old), high intensity (70%), and long duration (14 days) of feed restriction affected production performance and the plasma hormone in broilers (15). A previous study indicated that feed restriction can influence the liver organ index in broilers (16), but the effect is vague. For instance, 1 h of feeding and 3, 5, or 7 h feed restriction show a low to zero impact on the relative weights of broilers from the 8^th^ to 28^th^ day of age when compared to a control group (17). Feed restriction during the first 12 weeks of life decreased the density of new neurons involved in neurogenesis in the hippocampal formation but did not affect the hippocampal volume and the total number of neurons (18). The hippocampus is related to functions of emotion and reaction, and is responsible for spatial learning ability and memory in birds (19). In chickens, it is additionally responsible for neuroplasticity (20) and altering dopaminergic components of the hippocampus (21). On the other hand, dopamine, a major catecholamine neurotransmitter in the central nervous system of mammals and birds, was known to be affected by learning ability, food intake behaviors, and feed restriction (22–24), and can modulate the hippocampal synaptic plasticity (25). That is, therefore, we think that dopamine may act in a role connecting behavior and neuron activity of hippocampal transcriptome in response to feed restriction. Figuring out the biological characterization of the slow- and fast-growing breeds on the development of organs, hormone secretion, and the hippocampus is essential, and would provide a better understanding of the process of domestication and artificial selection in domesticated chickens.

The study aimed to explore the changes in organ index, plasma dopamine, and transcriptome profile of the hippocampus between slow-growing dual-purpose chickens and fast-growing broiler strains in response to feed restriction. This study will shed light on to the different breeds' biological traits, with possible uses in improving breeding strategy and feed management.

## Materials and Methods

### Animals and Treatments

The experimental protocols were approved by the China Agricultural University Laboratory Animal Welfare and Animal Experimental Ethical Inspection Committee (approval number: CAU20180619-5). One hundred healthy 1-day-old female Weining dual-purpose chicks were provided by Yuansheng Animal Husbandry Co., Ltd., China This is a heritage, slow-growing (11 g/d growth rate) breed. One hundred 1-day-old female Jinlinghua broiler chicks were provided by Nanning Jinlinghua Agriculture and Animal Husbandry Group Co., Ltd., China. This is a modern, fast-growing (27 g/d growth rate) breed. All birds were reared in a brooding barn using two enclosures (0.50 × 0.50 × 0.30 m; one for each breed), respectively. The temperature was kept above 32°C from post-hatching to 16 days old. Thereafter, the temperature was gradually decreased to room temperature. At the age of 27 to 29 days, each bird was moved to a single cage (0.19 × 0.30 × 0.40 m) constructed on all sides with wire mesh. Birds were numbered with two 16 mm diameter foot rings on each leg and adapted to new environmental conditions for feed restriction program preparedness. Each cage had a feeder, drinker, perch, and droppings board. All chickens were randomly allocated to *ad libitum* or control feeding regimes to achieve a balanced sample size in each combination of breed and feeding regime. The treatments were therefore slow-growing Weining chickens fed *ad libitum* (SA, *n* = 50) or under feed restriction (SR, *n* = 50), as well as fast-growing Jinlinghua chickens fed *ad libitum* (FA, *n* = 50) or under feed restriction (FR, *n* = 50). Slow- and fast-growing chickens in the feed-restricted group were restricted from the age of 30 days to 60 days [the feed of the feed-restricted group was 70% of that of the control (15)]. The *ad libitum* measures were referred to in our previous study using the same breed and cage rearing (26). The amount of feed and the leftover feed of each chick was recorded daily.

### Organ Index

At 61 days, 10 randomized birds in each group were humanely slaughtered. The weights of the body, heart, liver, spleen, and brain were immediately collected and weighted by electrical scale (quantitative analysis at 0.01 g level). Organ index was calculated by formula as follows:


$$\begin{array}{l} Organ\;index=\frac{organ\;weight}{body\;weight}\;\times 100\% \end{array}$$

### Plasma Dopamine

At 61 days, plasma samplings were immediately collected from the abovementioned 10 birds in each group and placed in an anticoagulation tube, 4000 g centrifuge for 5 min at 4°C, then stored in 1.5 ml tubes at −20°C to prepare them for the subsequent dopamine detection. The concentrations of dopamine were then detected by an enzyme-linked immune sorbent assay kit (FU-Q411, China).

### Transcriptome Profile Analysis

At 61 days, the hippocampus of eight birds was immediately collected from the abovementioned 10 birds in each group and stored in the dry ice, and then at −80°C until further processing. Total RNA was extracted from the tissue using TRIzol® Reagent (Invitrogen) according to the manufacturer's instructions, and genomic DNA was removed using DNase I (TaKara). Then RNA quality was determined by a 2100 Bioanalyser (Agilent) and quantified using the ND-2000 (NanoDrop Technologies). Only high-quality RNA samples were used to construct a sequencing library.

RNA-seq transcriptome library was prepared following TruSeq^TM^ RNA sample preparation Kit from Illumina (San Diego, CA) using 1 μg of total RNA. Shortly, messenger RNA was isolated according to poly-A selection method by oligo (dT) beads and then fragmented by a fragmentation buffer. Next, double-stranded cDNA was synthesized using a SuperScript double-stranded cDNA synthesis kit (Invitrogen, CA) with random hexamer primers (Illumina). Then the synthesized cDNA was subjected to end-repair, phosphorylation, and “A” base addition according to Illumina's library construction protocol. Libraries were size selected for cDNA target fragments of 200-300 bp on 2% Low Range Ultra Agarose, followed by PCR amplified using Phusion DNA polymerase (NEB) for 15 PCR cycles. After being quantified by TBS380, the paired-end RNA-seq sequencing library was sequenced with the Illumina HiSeq X ten/NovaSeq 6000 sequencer (2 × 150 bp read length).

The raw paired-end reads were trimmed and quality controlled by SeqPrep (https://github.com/jstjohn/SeqPrep) and Sickle (https://github.com/najoshi/sickle), with default parameters. Then clean reads were separately aligned to the reference genome with the orientation mode using TopHat (http://tophat.cbcb.umd.edu/, version2.0.0) (27) software. The mapping criteria used in Bowtie were as follows: sequencing reads should be uniquely matched to the genome allowing up to two mismatches, without insertions or deletions. Then, the region of the gene was explored according to different site depths and the operon was obtained. In addition, the whole genome was split into multiple 15k bp windows that each share an overlap of 5k bp. New transcribed regions were defined as more than two consecutive windows without the overlapped region of the gene, where at least two reads mapped per window were in the same orientation.

To identify DEGs (differentially expressed genes) between two different samples, the expression level of each transcript was calculated according to the fragments per kilobase of exon per million mapped reads (FRKM) method. RSEM (28) was used to quantify gene abundances. R statistical package software EdgeR (Empirical Analysis of Digital Gene Expression in R) (29) was utilized for differential expression analysis. In addition, functional-enrichment analysis, including Gene Ontology (GO) and Kyoto Encyclopedia of Genes and Genomes (KEGG), was performed to identify which DEGs were significantly enriched in GO terms and metabolic pathways at Bonferroni-corrected *P*-value ≤ 0.05 compared with the whole-transcriptome background. GO functional enrichment and KEGG pathway analysis were carried out by Goatools (https://github.com/tanghaibao/Goatools) and KOBAS (http://kobas.cbi.pku.edu.cn/home.do) (30).

### Statistical Analysis

All data were analyzed and tested throughout IBM SPSS Statistics 21. The organ index has not met the assumptions for parametric analysis, and therefore has been analyzed via a non-parametrical method. Two-way non-parametrical ANOVA analysis, specifically the Scheirer-Ray-Hare test, was used for statistical analysis, and then the Kruskal-Wallis test (K-W test) was used for pairwise analysis. The main effects were analyzed when they showed no significant or interaction effects, whereas the simple effects were analyzed when the main effects and interaction effects were both significant. Plasma dopamine concentration was checked for normality and homogeneity of variance and meets the assumptions for parametric analysis, and was analyzed using a two-way ANOVA. The Duncan test was used in the Postdoc testing. All values with *P* < 0.05 were regarded as statistically significant.

## Results

### Organ Index

Organ indexes are shown in Table 1. The brain organ index was affected by both breed and feeding regime, but not by the interaction of breed and treatment. The brain organ index was higher in slow-growing dual-purpose chickens than in the fast-growing broiler breed, and higher in the *ad libitum* group than the feed restricted group (*P* < 0.05). The organ index of the heart, liver, and spleen showed no difference between slow- and fast-growing breeds, or between *ad libitum* and feed restriction treatments.

**Table 1:** Effects of feed restriction on organ index between slow- and fast-growing chickens.

**Effects**	**Factors**	**Group**	**Heart**	**Liver**	**Spleen**	**Brain**
Main effects	Breed	Slow-growing	0.0060 ± 0.0005	0.0155 ± 0.0021	0.0025 ± 0.0009	0.0032 ± 0.0006^a^
		Fast-growing	0.0064 ± 0.0012	0.0142 ± 0.0017	0.0024 ± 0.0010	0.0014 ± 0.0002^b^
	Treatment	*Ad libitum*	0.0062 ± 0.0009	0.0150 ± 0.0021	0.0025 ± 0.0010	0.0026 ± 0.0012^A^
		Feed restriction	0.0062 ± 0.0009	0.0148 ± 0.0019	0.0025 ± 0.0009	0.0020 ± 0.0008^B^
Interaction effect	Breed × Treatment *P*-value		0.189	0.136	0.667	0.334
Simple effects	Breed	SA	0.0059 ± 0.0005	0.0160 ± 0.0020	0.0024 ± 0.0007	0.0037 ± 0.0003
		FA	0.0065 ± 0.0010	0.0139 ± 0.0017	0.0025 ± 0.0013	0.0015 ± 0.0002
		SR	0.0061 ± 0.0005	0.0150 ± 0.0021	0.0026 ± 0.0010	0.0028 ± 0.0003
		FR	0.0062 ± 0.0013	0.0145 ± 0.0017	0.0024 ± 0.0007	0.0012 ± 0.0002
	Treatment	SA	0.0059 ± 0.0005	0.0160 ± 0.0020	0.0024 ± 0.0007	0.0037 ± 0.0003
		SR	0.0061 ± 0.0005	0.0150 ± 0.0021	0.0026 ± 0.0010	0.0028 ± 0.0003
		FA	0.0065 ± 0.0010	0.0139 ± 0.0017	0.0025 ± 0.0013	0.0015 ± 0.0002
		FR	0.0062 ± 0.0013	0.0145 ± 0.0017	0.0024 ± 0.0007	0.0012 ± 0.0002

a, b *represents the significant difference between slow- and fast-growing breeds in the same treatment*.

A, B *represents the significant difference between ad libitum vs. feed restriction in the same breed. SA, slow-growing dual-purpose chickens ad libitum; SR, slow-growing dual-purpose chickens feed restriction; FA, fast-growing broilers ad libitum; FR, fast-growing broilers feed restriction*.

### Plasma Dopamine

The plasma dopamine was affected by feed restriction and the interaction of breed and treatment (*P* < 0.05, Table 2), but not by breed alone. In the feed restricted group, the concentration was higher than that of the *ad libitum* group (*P* < 0.05, Table 2). The dopamine concentration in the SR group and FR group was higher than that in the SA group and the FA group, accordingly (*P* < 0.05, Table 2).

**Table 2:** Effects of feed restriction on plasma dopamine concentration between slow- and fast-growing chickens.

**Effects**	**Factors**	**Group**	**Concentrations**
Main effect	Breed (pooled)	Slow-growing breed	663.7 ± 96.1
		Fast-growing breed	677.0 ± 204.5
	Treatment (pooled)	*ad libitum*	566.5 ± 111.8^B^
		Feed restriction	774.3 ± 125.0^A^
Interaction effect	Breed × Treatment		
	*P*-value		0.028
Simple effect	Breed	SA	605.8 ± 76.8
		FA	527.2 ± 131.8
		SR	721.7 ± 78.9
		FR	826.8 ± 144.7
	Treatment	SA	605.8 ± 76.8^B^
		SR	721.7 ± 78.9^A^
		FA	527.2 ± 131.8^B^
		FR	826.8 ± 144.7^A^

A, B *represents the significant difference between ad libitum vs. feed restriction in the same breed. SA, slow-growing dual-purpose chickens ad libitum; SR, slow-growing dual-purpose chickens feed restriction; FA, fast-growing broilers ad libitum; FR, fast-growing broilers feed restriction*.

### Hippocampal Transcriptome Profile Analysis

The sequenced data (clean reads) after quality control were compared with the reference genome, and the data obtained from each group were averaged. It can be seen that the mapping rate between the sequenced data and the reference genome was 88.67%.

**Figure 1:**
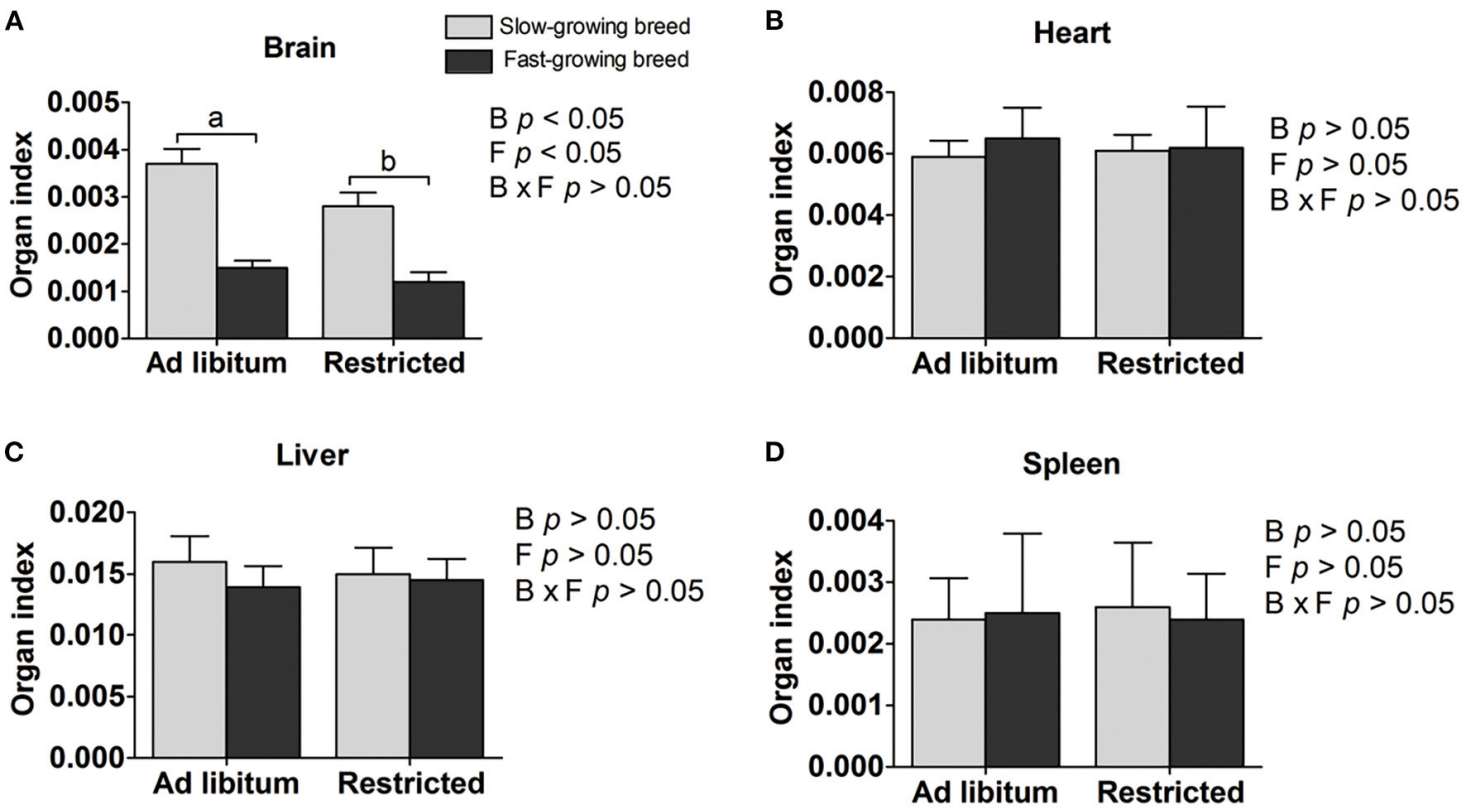
Organ indexes of brain **(A)**, heart **(B)**, liver **(C)**, and spleen **(D)** between slow-growing and fast-growing breed birds. SA, slow-growing dual-purpose chickens *ad libitum*; SR, slow-growing dual-purpose chickens feed restriction; FA: fast-growing broilers *ad libitum*; FR, fast growing broilers feed restriction. a, b means the significant difference between ad libitum vs feed restriction in the same breed.

After the feed restriction treatment, the Venn diagram formed by the data processing of each group is shown below (Figure 1). The SA and SR groups had 11930 genes co-expressed, and 967 and 135 genes specifically expressed. The FA and FR groups had 13,383 co-expressed genes, and 172 and 768 genes specifically expressed. The SA and FA groups have 12,665 genes co-expressed, and 232 and 890 genes specifically expressed. The SR and FR groups had 11,828 genes co-expressed, and 238 and 2,324 genes specifically expressed.

### Differently Expressed Genes

**Figure 2:**
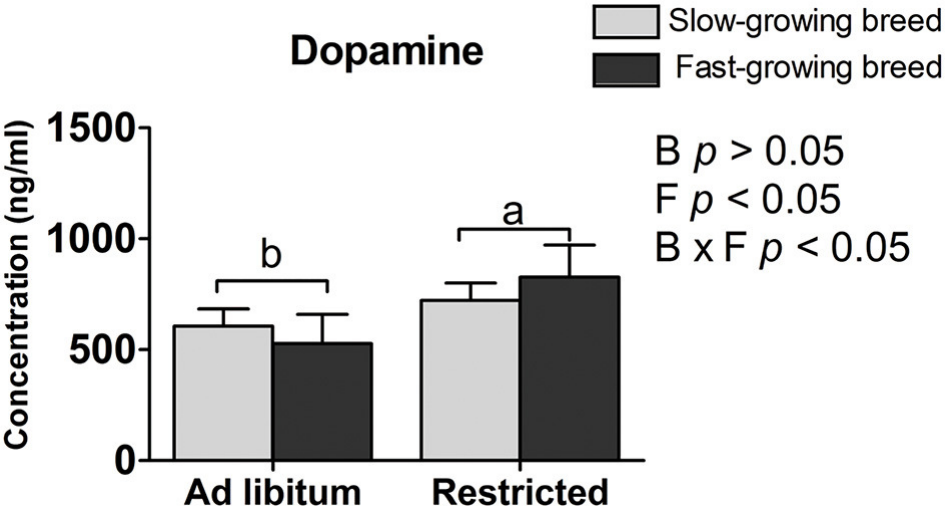
Differently expressed genes between groups. The discrepancy was more significant with |log2 fold change| < 1 and adjusted *P*-value < 0.05. SA, slow-growing dual-purpose chickens *ad libitum*; SR, slow-growing dual-purpose chickens feed restriction; FA, fast-growing broilers *ad libitum*; FR, fast-growing broilers feed restriction. a, b means the significant difference between *ad libitum* vs feed restriction in the same breed.

After the feed restriction treatment, the gene expression levels of each group were significantly adjusted up and down as shown below (|log2 fold change| < 1, and adjusted *P* < 0.05) (Figure 2). Compared with the SA group, the SR group was upregulated in 84 genes and downregulated in 62 genes. As compared with the FA group, the FR group was upregulated in 701 genes and downregulated in 521 genes. Compared with the SA group, the FA group was upregulated 475 genes and downregulated 964 genes. As compared with the SR group, the FR group was upregulated in 2,647 genes and downregulated in 2,389 genes.

### Gene Ontology Enrichment Analysis

**Figure 3:**
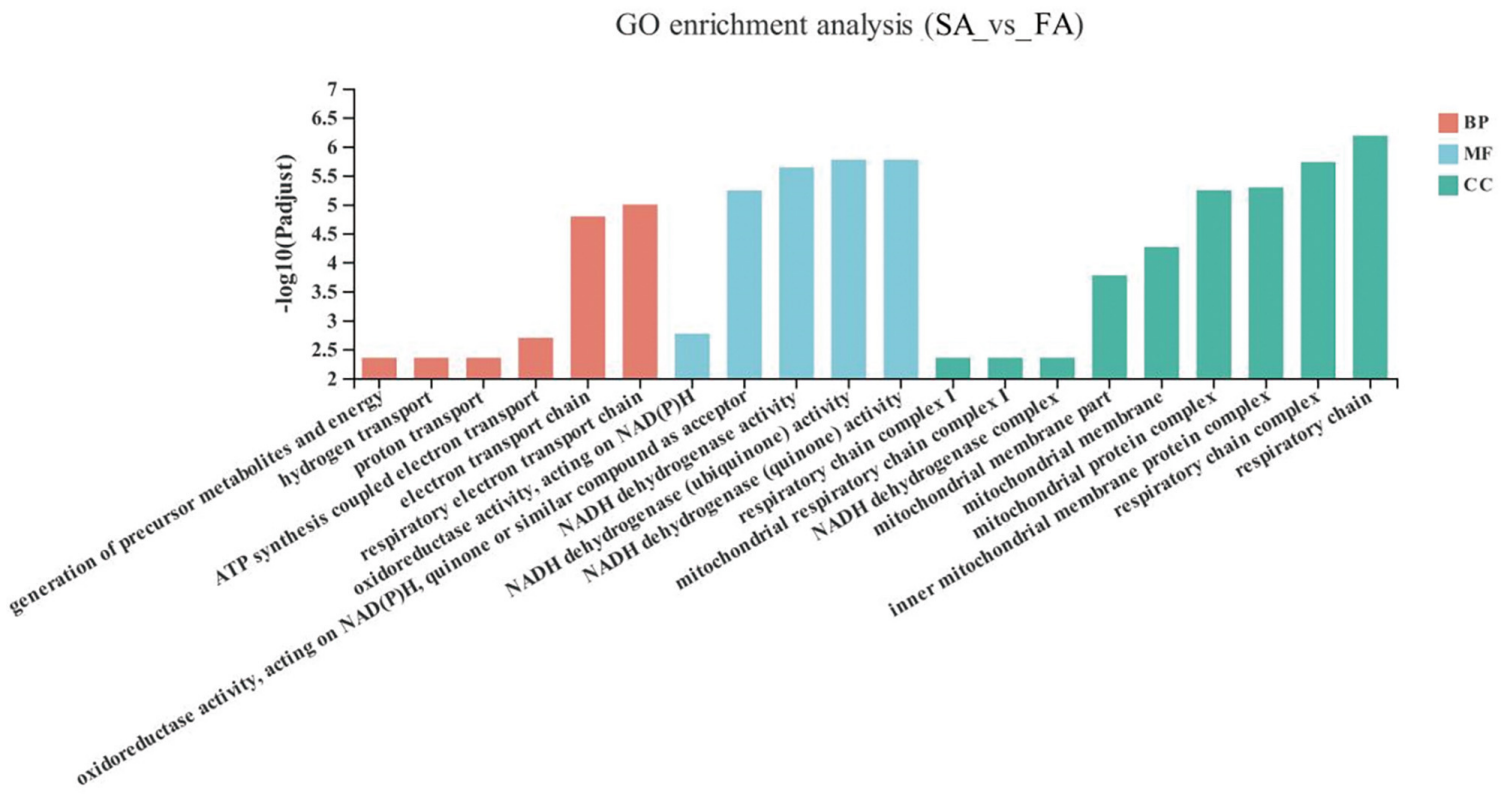
GO terms of differentially expressed genes (DEGs) between SA and FA groups. SA, slow-growing dual-purpose chickens *ad libitum*; FA, fast-growing broilers *ad libitum*.

The GO enrichment analysis results of the significant up-down genes between the SA and FA groups are shown in Figure 3. The DEGs in the SA and FA groups were significantly enriched in pathways related to the mitochondrion and respiratory electron transport chains. With respect to the biological functions: respiratory electron transport chains, electron transport chains, ATP synthesis coupled electron transport, proton transport, hydrogen transport, and generation of precursor metabolites and energy were enriched between groups (*P* < 0.05). For the molecular function: NADH dehydrogenase (quinone) activity; NADH dehydrogenase (ubiquinone) activity; NADH dehydrogenase activity; oxidoreductase activity, acting on NAD(P)H, quinone or similar compound as acceptor; and oxidoreductase activity, acting on NAD(P)H were found to be significantly enriched (*P* < 0.05). Regarding the cellular component: respiratory chain, respiratory chain complex, inner mitochondrial membrane protein complex, mitochondrial protein complex, mitochondrial membrane, mitochondrial membrane part, NADH dehydrogenase complex, mitochondrial respiratory chain complex I, and respiratory chain complex I were differently enriched (*P* < 0.05).

**Figure 4:**
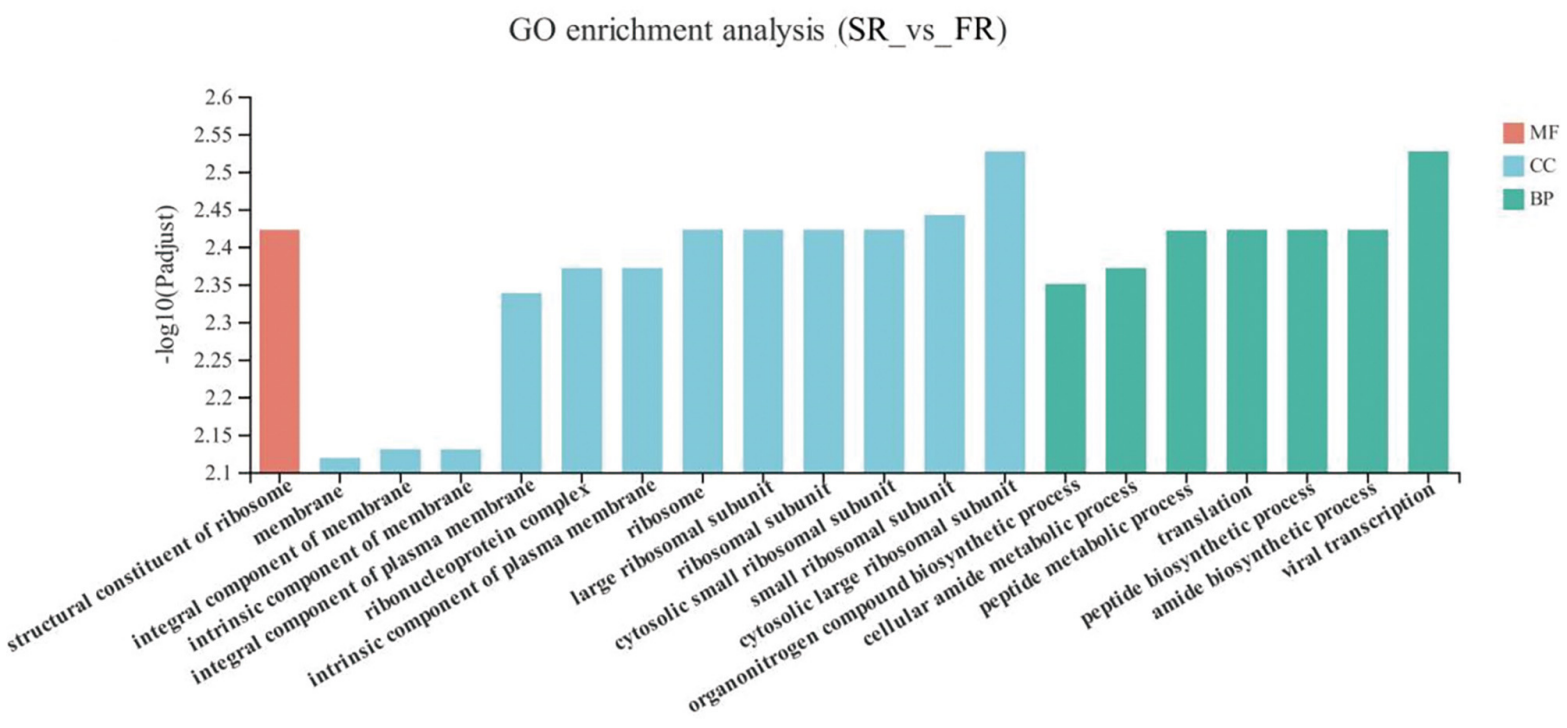
GO terms of differentially expressed genes (DEGs) between SR and FR groups. SR, slow-growing dual-purpose chickens feed restriction; FR, fast-growing broilers feed restriction.

The DEGs between the SR group and the FR group enriched in GO as displayed in Figure 4. With respect to biological function, DEGs were mainly enriched in pathways related to Amide biosynthetic process and cellular amide metabolic process (*P* < 0.05). The structural constituent of ribosome was enriched in the molecular function (*P* < 0.05). Membrane and ribosomes were enriched in the cellular component (*P* < 0.05).

### Kyoto Encyclopedia of Genes and Genomes Enrichment Analysis

**Figure 5:**
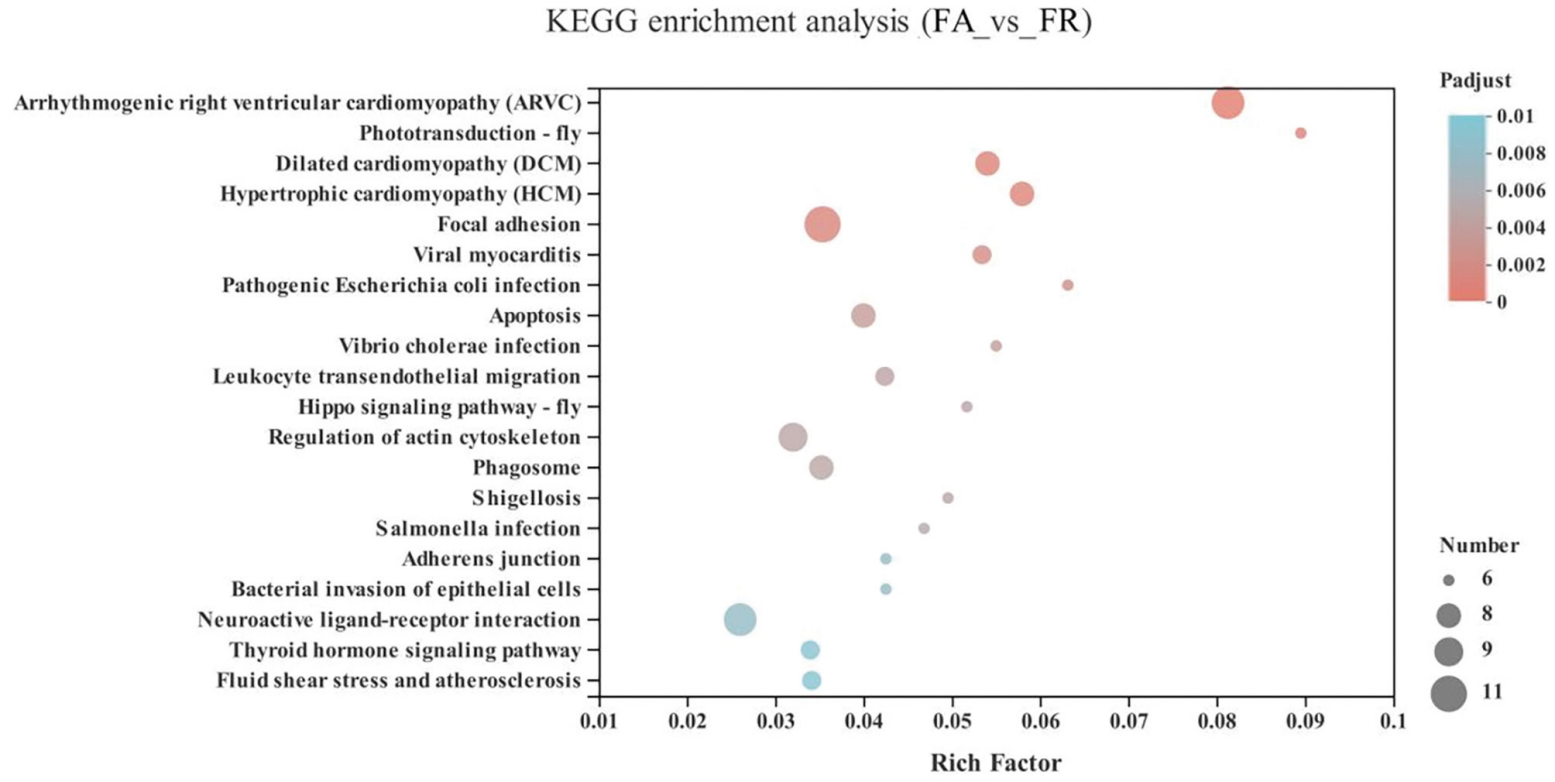
KEGG pathway enrichment analysis of differentially expressed genes (DEGs) between SA and FR groups. SA, slow-growing dual-purpose chickens *ad libitum*; FR, fast-growing broilers feed restriction.

Compared with the FA group, the significantly upregulated genes in the FR group were mainly enriched in the cardiovascular diseases category, including arrhythmogenic right ventricular cardiomyopathy (ARVC) (10 genes), dilated cardiomyopathy (DCM) (8 genes), hypertrophic cardiomyopathy (HCM) (8 genes), viral myocarditis (7 genes), fluid shear stress and atherosclerosis (7 genes), as well as neurodegenerative diseases, including neuroactive ligand-receptor interaction (*GALR1, HTR5A, GLP1R, VIPR1, NPB*, ENSGALG00000038742, ENSGALG00000039810, ENSGALG00000045754, ENSGALG00000040736, and ENSGALG00000040953) (*P* < 0.05) (Figure 5 and Supplementary Table 1).

**Figure 6:**
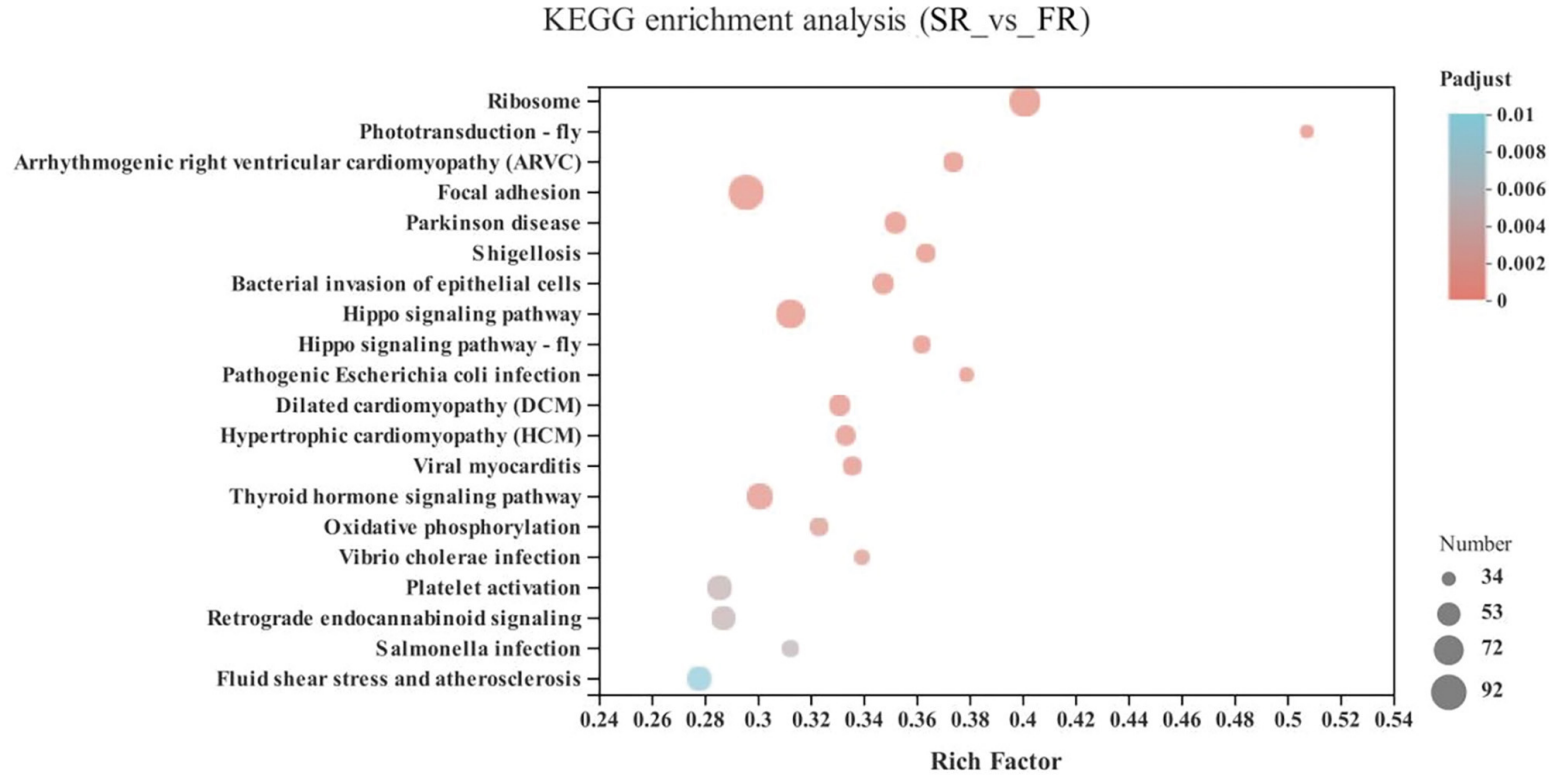
KEGG pathway enrichment analysis of differentially expressed genes (DEGs) between SA and FA groups. SA, slow-growing dual-purpose chickens *ad libitum*; FA, fast-growing broilers *ad libitum*.

In the energy metabolism category of oxidative phosphorylation (*MT-ND3, MT-ND2, MT-CO1, ND6, MT-ND4L, MT-ATP8, MT-ND4, ND5, COX3, MT-CYB, ND1, ATP6V1G3, MT-CO2*, and *ATP6*), the neurodegenerative diseases category, including Parkinson disease (*MT-ND3, MT-ND2, MT-CO1, ND6, MT-ND4L, MT-ND4, ND5, COX3, MT-CYB, ND1, MT-CO2*, and *ATP6*) and Huntington disease, and the retrograde category of the nervous system related to endocannabinoid signaling, were enriched in the SA and FA groups (*P* < 0.05) (Figure 6 and Supplementary Table 1).

**Figure 7:**
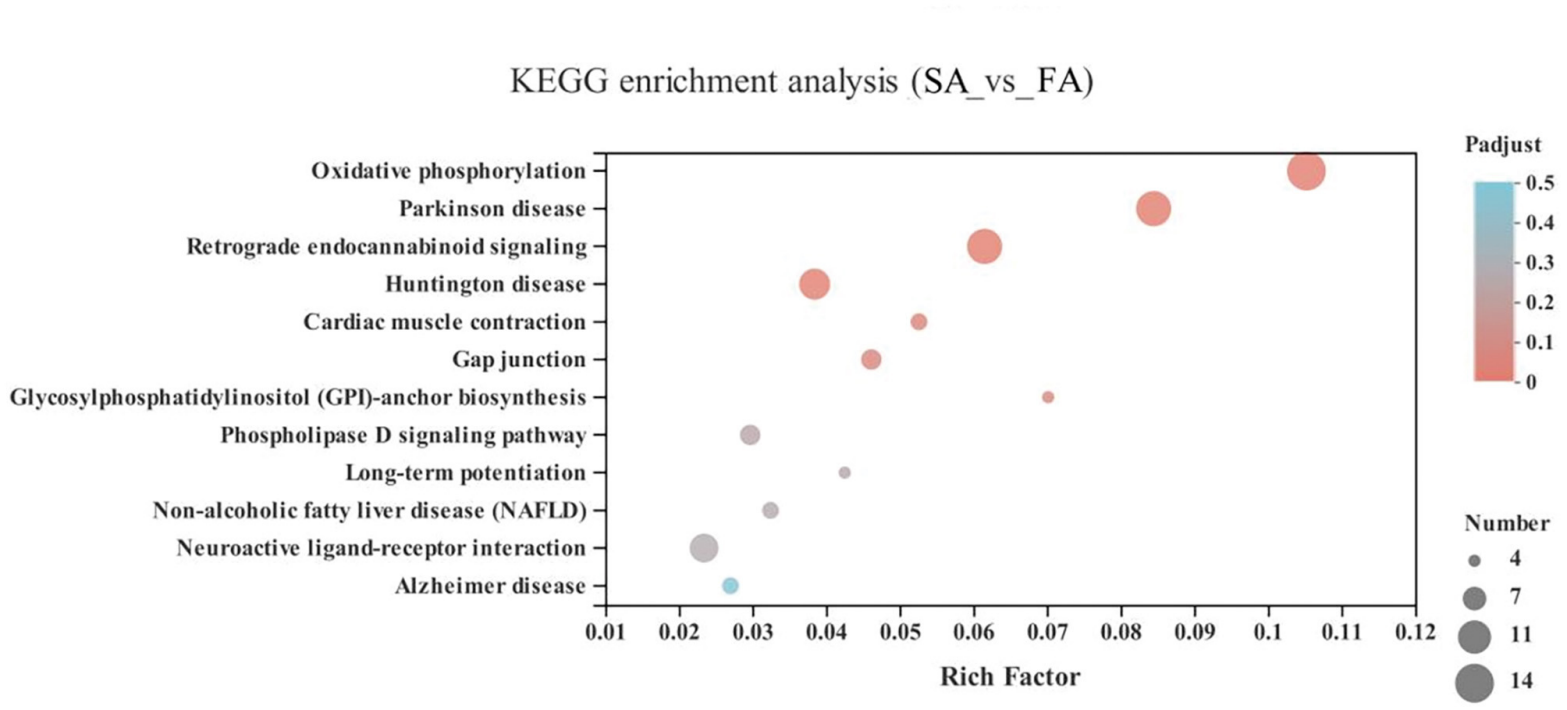
KEGG pathway enrichment analysis of differentially expressed genes (DEGs) between SR and FR groups. SR, slow-growing dual-purpose chickens feed restriction; FR, fast-growing broilers feed restriction.

Compared with the SR group, the FR group was enriched in pathways including arrhythmogenic right ventricular cardiomyopathy (ARVC) (46 genes), dilated cardiomyopathy (DCM) (49 genes), hypertrophic cardiomyopathy (HCM) (46 genes), viral myocarditis (44 genes), and fluid shear stress and atherosclerosis (57 genes), plus in neurodegenerative diseases category, including Parkinson disease (50 genes) and Alzheimer disease (51 genes), and lastly in the energy metabolism category of oxidative phosphorylation (43 genes) (*P* < 0.05) (Figure 7 and Supplementary Table 1).

## Discussion

Organs are the basic “facilities” of animal life processes and the material basis of their physiological functions, which directly affect the speed of weight gain, the health status of the animal, and even the adaptation to their environment of chickens (31). Organ index is a biological characteristic index, and the index size can reflect the workload of the organ in the organism to a certain extent (16, 17). In our study, the brain organ index showed sd higher in the *ad libitum* groups than the feed-restricted groups; these results are similar to the findings of a review which concluded that stress can reduce the volume of the brain during times of adversity in humans (32). Previous studies indicate that the highest relative liver weight was observed in the birds fed *ad libitum* compared to the feed restricted group when withdrawing the feed for 7 hours from 10 days to 30 days of age in broilers (16). The relative weight of the heart, liver, spleen, gizzard, pancreas, and intestine remained unaffected by the feed-restricted treatments from 8 to 28 days of age (17). However, feed restriction to 70% for a duration of 30 days had a limited effect on the heart, liver, and spleen organ index between slow- and fast-growing breeds in our study. Furthermore, the brain organ index displayed a greater increase in slow-growing dual-purpose chickens than in fast-growing broiler breeds, which might be supported by previous findings that artificial selection has altered the internal morphology of various animals, such as causing an overall decrease in brain size in mink (33). Besides, artificial selection has selected for large body size and pectoral muscles, rapid growth, and increased relative gut length in fast-growing broilers (34). The difference in brain organ index may be attributed to the process of intensive breeding and selection programs, which has been selected for a reduction of brain size (2, 35). In Red junglefowl, the brain size is reduced in response to artificial selection, and it is a tradeoff for other physiological traits, such as reproduction and growth (36).

The dopamine concentration in the feed restricted group was higher than the *ad libitum* group. Our result may be supported by that previous finding that the intracerebroventricular injection of dopamine decreases food intake in chickens (37), and further confirms the association of dopamine with feed intake (22, 23). One reason may be that feed restriction stimulates a greater state of excitement in the birds when feeding owing to their greater hunger. The mechanism is similar to findings that dopamine hyperfunction provides an abnormal driving force in afflicted patients that causes mental excitement (38). To some extent, increased dopamine levels are associated with social anxiety disorder (39, 40). That is, too much dopamine and dopamine hyperfunction lead to irritability, which results in emotional dysfunction, nerve reflex rapid reaction, and extreme hyperactivity (41). In addition, dopamine was influenced by the interaction of breed and treatment. Similarly, the higher dopamine varieties have other functions affecting the process of hyperfunction and hyperactivity, which may be responsible for the physiological process between the SR and SA groups, as well as the FR and FA groups.

When it comes to gene expression of the hippocampus, none of DEGs were enriched in the GO term and KEGG pathways. A previous study indicated that a 12-week duration of feed restriction reduces hippocampal neurogenesis and causes potential chronic stress, but is not of consequence to health outcomes in birds (18). Feed restriction in our study lasting 4 weeks has shown significant impacts on the hippocampal transcriptome, and a greater breed effect in the fast-growing breed than slow-growing breed. The greater number of DEGs, GO terms and functional KEGG pathways may implicate the increased plasticity of gene expressions in the fast-growing breed compared with the slow-growing breed in response to feed restriction (11). Prior studies showed that slow- and fast-growing breeds had a different transcriptome profile in the breast muscle (42) and leg muscle (9), which may mirror the transcriptome profile between breeds in our study. Thus, the gene expression pattern is greatly affected due to feed restriction, which has also demonstrated that the hippocampus is a sensitive area easily influenced by external stimuli (18).

Compared with the FA group, the significantly upregulated genes in the FR group were mainly enriched in the cardiovascular disease category, including arrhythmogenic right ventricular cardiomyopathy (ARVC), dilated cardiomyopathy (DCM), hypertrophic cardiomyopathy (HCM), and viral myocarditis. Accordingly, fast-growing broilers are highly susceptible to stress-induced cardiac arrhythmia (43). Cardiovascular diseases may cause high mortality rates, recognized as sudden death syndrome (44). In addition, dopamine is a precursor to norepinephrine in noradrenergic nerves and in certain areas of the central nervous system involved in the cardiovascular system (45), which may support the higher concentration of dopamine in the FR group than the FA group in response to cardiovascular function. Also, DEGs in the FR group were enriched in the neuroactive ligand-receptor interaction pathway of the neurodegenerative disease category. A previous study indicated that feed restriction can decrease the density of neurogenesis in hippocampal formation in broilers (18). Previous results and our own seem to suggest that feed restriction is a detriment for neural-related development. Also, dopamine is related to hippocampal synaptic plasticity (25), cognitive functions (e.g., episodic memory, speed, fluency), and facilitating the responsivity of divergent neural networks (46). Thus, the higher dopamine concentration in the FR group than the FA group may be connected to the neural-related development in the hippocampus in fast-growing broilers.

The upregulated DEGs of FA were enriched in Parkinson's disease and Huntington's disease compared to the SA group. It is possible that their accelerated breeding speed gives the fast-growing broilers increased potential to develop neural and pharmacological problems (4). Besides, oxidative phosphorylation in the energy metabolism category in the FA group was higher than in the SA group. Oxidative phosphorylation is associated with the oxidoreductase chain in the mitochondria, which implicated that slow- and fast-growing breeds had different energy metabolism abilities and trade-offs in energy allocation (2, 31). Additionally, the GO terms, biological function, molecular function, and cellular component process were enriched in the mitochondria, respiratory chain, and energy metabolism. It is accepted that differences in metabolic rate, oxidative stress ability, and energy metabolism arise in response to the different purposes of artificial selection (11, 31).

As when we compared FR to FA, the cardiovascular diseases category, including arrhythmogenic right ventricular cardiomyopathy (ARVC), dilated cardiomyopathy (DCM), hypertrophic cardiomyopathy (HCM), viral myocarditis, fluid shear stress, and atherosclerosis pathways, were found between the comparisons of FR to SR. Thus, it seems that feed restriction is likely linked to cardiovascular diseases, illustrating that feed restriction to 70% for 30 days is more detrimental in the fast-growing breed than the slow-growing breed. Similarly, more DEGs were enriched in the Parkinson's disease and Alzheimer's disease areas of the neurodegenerative diseases category. The hippocampus is a sensitive brain area, which is influenced by Alzheimer's disease, epilepsy, and depression (47). Parkinson's disease and Alzheimer's disease were critically implicated by dopamine (48). Unexpectedly, the dopamine did not differ between SR and FR groups, for which we speculate that a significant difference in dopamine levels may not necessarily lead to those diseases, and this requires further study in different breeds. In the GO terms, it seemed that DEGs were enriched in the membrane and ribosome-related processes; the reason for this was unclear and requires further investigation. All in all, we conclude that the fast-growing breed is more susceptible to the detrimental effects of feed restriction. Further study should be focused on the validation of the key genes involved and the role of these genes on related pathways. Besides, the mechanism of dopamine in connecting feeding behavior and genetic aspects requires further investigation, especially for the classification of the role of dopamine on feeding behavior and emotion.

## Conclusion

Brain organ index was affected by feed restriction and breed. The Feed restricted group had greater dopamine hyperactivity than the *ad libitum* group in both slow- and fast-growing breeds. Differently expressed genes were enriched in the cardiovascular disease and neurodegenerative disease categories in the fast-growing breed, suggesting feed restriction to 70% for 30 days is a disadvantage for the fast-growing breed. Feed restriction had less effect on the hippocampal transcriptome profile in the slow-growing breed.

## Data Availability Statement

The datasets presented in this study can be found in online repositories. The names of the repository and accession numbers can be found below: https://www.ncbi.nlm.nih.gov/sra/, PRJNA745252.

## Ethics Statement

The animal study was reviewed and approved by China Agricultural University Laboratory Animal Welfare and Animal Experimental Ethical Inspection Committee (approval number: CAU20180619-5).

## Author Contributions

XZhao obtained the funding. CY, SC, and XZhao designed this project. CY, SC, WLiu, JX, ZL, HL, JL, XZhang, MO, ZC, and WLi performed the experiment. CY, WLiu, and SC analyzed and interpreted the data. CY, SC, and XZhao drafted and revised the manuscript. All authors agreed upon publication.

## Funding

This study received funding from the Guangdong Provincial Key Laboratory of Animal Molecular Design and Precise Breeding (2019B030301010), the Key Laboratory of Animal Molecular Design and Precise Breeding of Guangdong Higher Education Institutes (2019KSYS011), the Joint Fund of Basic and Applied Basic Research Fund of Guangdong Province (Number: 2019A1515110598), and the Joint Projects of GuizhouNayong Professor Workstation (Number: 201705510410352). The funder was not involved in the study design, collection, analysis, interpretation of data, the writing of this article or the decision to submit it for publication.

## Conflict of Interest

The authors declare that the research was conducted in the absence of any commercial or financial relationships that could be construed as a potential conflict of interest.

## Publisher's Note

All claims expressed in this article are solely those of the authors and do not necessarily represent those of their affiliated organizations, or those of the publisher, the editors and the reviewers. Any product that may be evaluated in this article, or claim that may be made by its manufacturer, is not guaranteed or endorsed by the publisher.
